# The Role of Low-Density Lipoprotein Receptor-Related Protein 1 in Lipid Metabolism, Glucose Homeostasis and Inflammation

**DOI:** 10.3390/ijms19061780

**Published:** 2018-06-15

**Authors:** Virginia Actis Dato, Gustavo Alberto Chiabrando

**Affiliations:** 1 Departamento de Bioquímica Clínica, Facultad de Ciencias Químicas, Universidad Nacional de Córdoba, Córdoba X5000HUA, Argentina; vickyactisdato@gmail.com; 2 Consejo Nacional de Investigaciones Científicas y Técnicas (CONICET), Centro de Investigaciones en Bioquímica Clínica e Inmunología (CIBICI), Córdoba X5000HUA, Argentina

**Keywords:** cholesterol, endocytosis, lipoprotein, membrane, traffic

## Abstract

Metabolic syndrome (MetS) is a highly prevalent disorder which can be used to identify individuals with a higher risk for cardiovascular disease and type 2 diabetes. This metabolic syndrome is characterized by a combination of physiological, metabolic, and molecular alterations such as insulin resistance, dyslipidemia, and central obesity. The low-density lipoprotein receptor-related protein 1 (LRP1—A member of the LDL receptor family) is an endocytic and signaling receptor that is expressed in several tissues. It is involved in the clearance of chylomicron remnants from circulation, and has been demonstrated to play a key role in the lipid metabolism at the hepatic level. Recent studies have shown that LRP1 is involved in insulin receptor (IR) trafficking and intracellular signaling activity, which have an impact on the regulation of glucose homeostasis in adipocytes, muscle cells, and brain. In addition, LRP1 has the potential to inhibit or sustain inflammation in macrophages, depending on its cellular expression, as well as the presence of particular types of ligands in the extracellular microenvironment. In this review, we summarize existing perspectives and the latest innovations concerning the role of tissue-specific LRP1 in lipoprotein and glucose metabolism, and examine its ability to mediate inflammatory processes related to MetS and atherosclerosis.

## 1. Introduction

The low-density lipoprotein receptor-related protein 1 (LRP1 or CD91) is a type I transmembrane protein belonging to the low-density lipoprotein receptor (LDL-R) family [1]. This receptor is principally expressed in neurons, epithelial and muscle cells, fibroblasts, retinal Müller glial cells, monocytes, macrophages, hepatocytes, adipocytes, vascular smooth muscle cells (VSMCs), and tumor cells [1,2,3]. LRP1 is synthesized as a precursor glycoprotein of 600 kDa, and processed by furin in the middle Golgi network to produce two subunits: (i) the extracellular α-subunit of 515 kDa, containing four extracellular ligand-binding domains; and (ii) the transmembrane and intracellular β-subunit of 85 kDa that possesses the YxxL, di-Leucine (LL), and NPxY motifs, which are essential for endocytosis and intracellular signaling activation by LRP1 [1].

LRP1 binds and internalizes more than 40 unrelated ligands, such as the α2-macroglobulin-protease complex (α2M) and triglyceride-rich lipoprotein-derived apolipoprotein E (apoE), which are mainly degraded by lysosomas [4]. In addition, the endocytosis and intracellular trafficking of LRP1 plays a key role in regulating the cellular functions and activities of other receptors and plasma membrane proteins that molecularly interact with LRP1, such as platelet-derived growth factor receptor β (PDGFR β) [5], urokinase-plasminogen activator receptor (uPAR) [6], membrane type 1-MMP (MT1-MMP) [7], β1-integrin [8,9], insulin receptor (IR) [10,11], insulin-like growth factor receptor-1 (IGFR-1) [12] and glucose transporter type 4 (GLUT4) [13].

Metabolic syndrome (MetS) is a highly prevalent disorder characterized by a combination of metabolic alterations, including dyslipidemia, impaired glucose homeostasis, and an exacerbated inflammatory process, which lead to an increased risk of type 2 Diabetes Mellitus (T2DM) and cardiovascular disease (CVD) [14]. Based on its ability to exert complex and multi-molecular extra- and intracellular functions, LRP1 has been shown to be involved in MetS. In this way, this receptor plays a key role in the uptake and processing of modified and non-modified lipoproteins in different tissues, which is associated with the promotion of intracellular lipid accumulation occurring during dyslipidemia [15,16,17]. In glucose homeostasis, LRP1 has a regulatory action on the insulin receptor (IR) and GLUT4 activation [10,16,18], which has been strongly associated with insulin resistance developed during MetS. In addition, inflammatory processes induced during MetS are also involved in the development of atherosclerosis [19]. In this case, LRP1 is a molecular and cellular mediator for M2 to M1 macrophage transformation and production of proinflammatory factors that occur in the development of atherosclerotic lesions [20].

At the level of LRP1 gene expression, this receptor may be regulated by peroxisome proliferator receptor activated-γ (PPARγ), as a result of the presence of the peroxisome proliferator response element (PPRE) in the LRP1 promoter region [21]. PPARγ is a nuclear receptor which can regulate important proteins and enzymes that are involved in glucose metabolism control, such as the glucose transporters GLUT2 and GLUT4 [22]. Rosiglitazone (RGZ) is a potent PPARγ agonist with anti-diabetic properties, but it has also been associated with several cardiovascular risks [23]. Some studies have indicated that RGZ can modulate LRP1 expression by targeting PPARγ expression in cell culture models (including the HepG2 cell line), but only in a dose-dependent manner, since RGZ low doses (<5 μM) up-regulate the LRP1 expression, whereas higher doses (>5 μM) can produce LRP1 downregulation [24]. Related to this, genetic studies have shown an association of the T-allele form of the exon 3′ *LRP1* gene with the development and progression of MetS [25]. In genome-wide association studies (GWAS), it was also found that the LRP1 single nucleotide polymorphism (SNP), rs4759277, is strongly linked to phenotypic alterations of the carbohydrate metabolism, such as fasting insulin, C-peptide and homeostasis assessment of insulin resistance [26].

Although several studies have reported that LRP1 is involved in MetS, exactly how this receptor participates in these altered metabolic regulations is still not well understood. In this review, we report on the current understanding of the potential role of tissue-specific LRP1 in lipid and glucose metabolism control along with examining its ability to mediate inflammatory processes with respect to the development of MetS.

## 2. LRP1 in Lipoprotein Uptake and Lipid Metabolism

Non-modified circulating lipoproteins bind to the specific LDL-R family members (Very low-density lipoprotein receptor (VLDL-R), apoE receptor 2 (apoE-R2), LDL-R, and LRP1) localized in the plasma membrane of several types of cells (principally hepatocytes, adipocytes, muscle cells and macrophages), which are internalized by endocytosis through a direct interaction with these receptors [27]. After endocytosis, the intracellular traffic of triglyceride-rich lipoproteins (TRLs) is more complex than the classical degradation pathway of low-density lipoproteins [28]. Once internalized, TRLs disintegrate into endosomes, which is followed by a differential sorting of TRL components. The core lipids and apolipoprotein B are targeted to lysosomes, while the TRL-derived apoE remains in the recycling endosomes [29]. Immunofluorescence studies have indicated that LRP1 mediates the accumulation of apoE in early (early endosome antigen 1 (EEA1)-positive) endosomes in human hepatoma cells [30], with the apoE processing being different from other LRP1 ligands such as receptor-associated protein (RAP) and activated α2-macroglobulin, which are directly targeted to lysosomal compartments [31]. apoE is trafficked to intracellular vesicles that contain high density lipoprotein (HDL) but not LRP1. Then, these vesicles containing TRL-derived apoE are mobilized by HDL-derived apoA I to be recycled to the plasma membrane, which is followed by apoE secretion and the formation of apoE-containing HDL. This event is accompanied by cholesterol efflux, revealing an intracellular link between TRL-derived apoE, cellular cholesterol transport, and the HDL metabolism where LRP1 is involved [30,32].

LRP1 is located in the basolateral membrane of the hepatocytes, where it participates in apoE-mediated uptake of triglyceride-rich lipoprotein remnants (chylomicrons (QM) and very low-density lipoproteins (VLDL)) from the plasma to the liver [33,34,35,36]. It has been demonstrated that insulin induces LRP1 translocation to the plasma membrane in liver, which was also related to an increase in the postprandial QM remnant uptake [37]. In contrast, when hepatic LRP1 expression was ablated, it was found that the QM remnant uptake was significantly reduced [37], indicating the preponderant dependency of this receptor on the QM metabolism to the hepatic level. Moreover, liver-specific LRP1 knockout mice have been characterized as being obese and having higher fasting glucose levels, lower glucose clearance, and liver steatosis, with the phenotypes being associated with decreased hepatic secretion of VLDL and attenuated insulin responses [16].

The participation of LRP1 in the regulation of the metabolism of intracellular triglycerides in adipocytes has also been demonstrated [38]. In this way, LRP1 is involved in the endocytosis of apoA-V, which generates a decreased triglyceride uptake in adipocytes that may be associated with increased lipolysis and energy expenditure, together with a reduced expression of lipid-associated proteins such as cidec and perilipin. Thus, these phenomena may have implications for the deregulation of lipogenesis and the development of obesity [38].

LRP1 is also involved in the accumulation and homeostasis of cholesterol in macrophages. Mice macrophages with selective LRP1 gene-deletion were found to contain significantly lower levels of total cholesterol than normal mice that expressed the LRP1 macrophage [39]. In addition, these animals also revealed elevated levels of triglycerides in plasma as a result of an increased accumulation of the triglyceride-rich lipoprotein particles in circulation. Nevertheless, this was a result of a defective catabolism of triglyceride-rich lipoprotein particles, since no increase in hepatic VLDL biosynthesis was found [39]. It was also reported that tyrosine phosphorylation of the NPxY motif of the LRP1 β-subunit initiated a signaling cascade along an LRP1/the adaptor protein Shc1/PI3K/Akt/PPARγ/Liver X receptor (LXR) axis, which down-stream mediated cellular cholesterol homeostasis in macrophages through the expression of the ATP binding cassette transporter A1 (ABCA1) [40]. 

Diverse studies have demonstrated that dyslipidemia has a deleterious impact on cardiac remodeling, with direct consequences on the extracellular matrix (ECM) components which can be degraded by cysteine proteases [41]. Enhanced VLDL concentrations induced cardiomyocyte intracellular cholesteryl ester (CE) accumulation in a LRP1-dependent manner [42]. In addition, this intracellular CE accumulation also increased cathepsin S protein levels, inducing altered structural and physical characteristics of secreted protoelastin [43]. Therefore, LRP1 can mediate the intracellular CE accumulation in cardiomyocytes, which could have an impact on pathological ventricular remodeling.

It has been reported that LRP1 avoids intracellular cholesterol accumulation by two mechanisms in different tissues. One of these is by the extracellular (α) subunit of LRP1 mediating transforming growth factor-β (TGF-β)-induced enhancement of Wnt5a, which promotes cholesterol export and inhibits cholesterol biosynthesis in mouse embryonic fibroblasts [44]. The second mechanism involves the cytoplasmic (β) subunit of LRP1, which regulates cholesterol accumulation through the interaction between the distal NPxY motif of this receptor and the serine/threonine kinase Erk2. This in turn, positively regulates the expression of ABCA1 and neutral cholesterol ester hydrolase (NCEH1), which together mediate the exportation and elimination of cellular cholesterol [44].

In conclusion, taken together, the above findings suggest that LRP1 may not only have an effect on cholesterol uptake, but also on its intracellular transport and metabolization in several cell types. Considering that LRP1 is a key factor in lipid homeostasis, this implies that altered functions of this receptor, including those that affect its intracellular trafficking, may promote cellular dysfunctions, principally in hepatocytes, adipocytes and macrophages, leading to the development of MetS.

## 3. LRP1 in the Metabolic Syndrome

### 3.1. LRP1 in the Tissue Processing of Modified Lipoproteins

Hyperlipidemia, a major MetS feature, induces inflammation and plays a fundamental role in atherosclerosis development [19]. This dyslipidemia is also associated with structural modifications in the native LDL that are currently recognized as a prerequisite for the initiation of lipid accumulation in the arterial intima [45]. Modified LDL can be generated by different mechanisms, including oxidation (oxLDL), glycation, alkylation, nitration and aggregation (aggLDL), or by an increased electronegative charge of the LDL particle (LDL(−)) [45]. Modified LDLs play a key role in atherogenesis and atherosclerosis progression, and induce atherosclerotic lesions through complex inflammatory and immunological mechanisms [19]. In the vascular wall, modified lipoproteins act as toxic compounds, thereby promoting foam cell activation, macrophage proliferation and migration, as well as excessive matrix extracellular remodeling by increased metalloproteinase (MMP) production [46,47,48,49]. The presence of modified LDLs in the vascular wall, together with pro-inflammatory cytokines, high levels of nitric oxide, and mechanical injury, can lead to apoptosis of VSMCs, which produces fibrous cap thinning and necrotic core formation, with calcification of the atherosclerotic plaques [50]. The adipose tissue also suffers the consequences of modified lipoproteins, since LDL(−) can induce adipose inflammation by promoting M1 transformation and infiltration of macrophages in fat tissue, which may explain the dysfunctionality of adipocytes in patients with MetS [51].

In general, modified LDLs can be bound and internalized by different cell surface receptors, such as scavenger receptors (LOX-1, scavenger receptor A (SRA), and CD36) and LRP1. The LRP1 receptor is the main one responsible for binding and internalizing aggLDL, whereas scavenger receptors can recognize other forms of modified LDLs [15,39,52]. However, LRP1 participation in the processing of other modified lipoproteins can not be discarded, since macrophages are able to incorporate diverse modified LDLs (mainly oxLDL), with LRP1 being necessary for these cells to become foam cells [53]. In contrast, the presence of aggLDL seems to be a key requirement for lipid accumulation in VSMCs, with LRP1 playing a fundamental role in lipid accumulation and VSMC-derived foam cell formation [15]. In this way, LRP1 is responsible for binding and internalizing aggLDL through a process that involves the participation of heparan sulfate proteoglycan (HSPG) in the cell surface of VSMCs [54]. In addition, it has been reported that aggLDL internalization in VSMCs may also require the activation of the P2Y purinoceptor 2 (P2Y2) receptors, actin cytoskeleton reorganization, and cell motility [55]. Finally, it was found that mice LRP1 deficiency in cardiomyocytes resulted in a reduced aggLDL uptake, thereby preventing left ventricular dysfunction, esterified cholesterol accumulation and insulin resistance [43].

### 3.2. LRP1 in Glucose Homeostasis Control

The cellular uptake of glucose requires specialized transporters that can mediate the ATP-independent facilitative diffusion process. These glucose transporters belong to a GLUT superfamily of proteins composed of fourteen members, which are grouped into three classes of GLUT (Class 1 to 3). Class 1 proteins (GLUTs 1–4) have been described as being those principally involved in whole-body glucose homeostasis [56]; while GLUT1 is expressed in many cell types, GLUT4 is mainly expressed in adipocytes and muscle cells [13]. Furthermore, in contrast with GLUT1, the GLUT4 activity is tightly regulated by insulin, which exerts a vital action on GLUT4 trafficking to the plasma membrane [57]. This disruption of GLUT4 translocation is the main cause of insulin resistance, and leads to an increased risk for developing of T2DM [13]. Finally, GLUT2 is the principal transporter of glucose in the plasma membrane in hepatocytes, whereas GLUT3 is the primary mediator of glucose uptake into neurons, with the effect of both these GLUT members on glucose control being either only partially dependent on, or independent of, insulin [58].

Numerous studies have reported that intracellular traffic of LRP1 is fundamental for regulating GLUT4 activity [13]. Related to this, the endocytic function and cell surface translocation of LRP1 is also modulated by insulin in various cell types, including cells that do not express GLUT4 [11,16]. At the same time, this LRP1 translocation is essential for the signaling activity of IR, as well as for GLUT4 expression in the plasma membrane [10,11]. In adipocytes and muscle cells, it has been shown that LRP1 is important for the formation and function of vesicles termed GSVs (for GLUT4 storage vesicles), which constitute the main compartments that store GLUT4 in these types of tissues [59]. From these GSVs, GLUT4 is transported and fused to the plasma membrane by the action of insulin, thereby increasing the expression of this transporter on the cell surface, where it binds, internalizes, and regulates the level of extracellular glucose [13,56]. It has been reported that LRP1 depletion in GSVs substantially affects the sorting of GLUT4 to the plasma membrane in 3T3-L1 adipocytes and adipose-specific LRP1 knock-out mice [59], with this event involving the insulin resistance and increased blood glucose levels occurring during MetS and T2DM [10,16]. Moreover, the reduced GLUT4 protein levels found in adipose cells of diabetic patients may be an early defect that contributes to the dysfunctional adipose tissue and associated alterations in adipokine secretion [60].

It has been reported that in astrocytes, LRP1 together with the scaffolding protein GIPC (GAIP-interacting protein, C terminus) mediate the molecular interaction between insulin-like growth factor 1 receptor (IGF1-R) and GLUT1. This allows GLUT1 to be retained inside the cell, modulating the brain glucose metabolism [12].

In hepatocytes, LRP1 can regulate the hepatic trafficking of GLUT2 to the plasma membrane by promoting an uptake of glucose after feeding and releasing gluconeogenesis-derived de novo glucose during fasting. However, exactly how LRP1 regulates GLUT2 trafficking is still not clear. It was found that liver-specific LRP1 gene deletion reduced the cell surface localization of GLUT2 in primary hepatocytes in the presence of insulin, suggesting a critical role for LRP1 in insulin-mediated GLUT2 translocation [16]. In addition, hepatic LRP1 depletion also resulted in defective insulin signaling, which included impaired phosphorylation of IR, Akt and glycogen synthase kinase 3-β (GSK3β), together with an incomplete suppression of gluconeogenic genes and significantly lower levels of IR expression at the plasma membrane. These findings strongly suggest that hepatic LRP1 is important for maintaining insulin sensitivity and glucose homeostasis, thereby preventing diet-induced steatosis, hyperglycemia, glucose intolerance, insulin resistance, and dyslipidemia.

Finally, an interaction between LRP1 and IR has been described in brain, which can regulate insulin signaling and glucose uptake in neurons [10]. In fact, neuronal LRP1 deficiency leads to a reduced IR localization in the plasma membrane, an impaired insulin signaling, and reduced levels of GLUT3 and GLUT4, which results in a reduced glucose uptake.

## 4. LRP1 in Atherosclerosis

It has been proposed that LRP1 is a key molecular factor that protects against the development and progression of atherosclerosis [44,61]. Nevertheless, other studies have paradoxically shown that the participation of this receptor is necessary for the formation of atherosclerotic lesions [15]. This ability to promote or suppress inflammatory processes that are involved in the early phases of atherosclerosis may explain the apparent dual action of LRP1. It is known that local inflammation of the artery wall is associated with macrophage recruitment at the level of the intima, thus contributing to the formation of atherosclerotic lesions [62]. Mice containing LRP1-deleted macrophages were shown to have a higher number of atherosclerotic lesions in the carotid and aorta [63], with these macrophages displaying an increased expression of proinflammatory cytokines, such as interleukin 1 β (IL-1β), interleukin 6 (IL-6) and tumor necrosis factor-α (TNF-α), concomitantly with a significant suppression of the PI3K/Akt survival pathway [64]. Thus, LRP1 may protect against atherosclerosis by attenuating inflammation through a decrease in the cell-surface abundance of the TNF receptor-1, and also by inhibiting the Iκ-B kinase/NF-κB intracellular activation. This attenuated inflammation mediated by LRP1 can also result in a reduced expression of other inflammatory mediators, such as inducible nitric oxide synthase and matrix metalloprotease-9 (MMP9) [1,20].

Various studies have suggested that LRP1 expressed in adipose tissues, in particular in the perivascular area surrounding the vessel wall, also plays a critical role in atherosclerosis development [65,66]. The perivascular adipose tissues (PVAT) of mice containing selective LRP1 deficiency in adipocytes were smaller but more inflamed with increased monocyte–macrophage infiltration and inflammatory gene expression, in comparison with mice expressing LRP1 in adipocytes [67]. Moreover, these adipocytes with LRP1 deficiency were dysfunctional, with the PVAT providing signals through the adventitia that modulated atherosclerotic lesion progression in response to hypercholesterolemia. However, other studies have shown that LRP1 may be an atheroprotective factor for the development of atherosclerosis, as it has been reported that mice macrophage LRP-1 deficiency increases cell death and inflammation by impairing phosphorylated Akt activation and efferocytosis [68].

Although the mechanisms concerning the functional role of LRP1 in efferocytosis in atherosclerosis are still not fully understood, it has been found that LRP1 integrates a multiprotein complex (composed of the receptor tyrosine kinase AXL and RAN-binding protein 9 RANBP9) which mediates dendritic cell (DC) efferocytosis and antigen cross-presentation [69]. This study revealed that AXL bound apoptotic cells (ACs), but required LRP1 to trigger internalization in murine CD8α+ DCs and human-derived DCs. These findings may have implications for future studies related to host defense, and for DC-based vaccines associated with the prevention of the development of atherosclerosis. In contrast to the LRP1 role in the development of atherosclerosis, a recent study has revealed that mice macrophage LRP-1 deficiency can also accelerate atherosclerosis regression, since regressing plaques showed a marked transition from M1 to M2 macrophage status, an enhanced reverse cholesterol transport, and an increased expression of CCR7, which promoted macrophage egress from atherosclerotic lesions [70].

The extracellular domain of LRP1 can be cleaved by proteases and released into the circulation [71,72], with the circulating form of LRP1 having been found at nano-molar concentrations in human plasma, and containing the α-subunit and a 55 kDa-fragment of the β-subunit, demonstrating that cleavage occurs close to the plasma membrane. Enzymes that mediate this process include the neuronal BACE1 protease and a hepatic metalloproteinase [73].

The soluble form of LRP1 (sLRP1) can bind and quench extracellular ligand interaction with the cellular LRP1, thereby regulating its intracellular trafficking or controlling several cell signaling pathways. In macrophages, sLRP1 causes the expression of TNF-α and the monocyte chemoattractant protein 1 (MCP-1) through activation of the mitogen-activated protein kinases (MAPK) and c-Jun N-terminal kinase (JNK) intracellular pathways [74]. In this way, LRP1 regulates macrophage activity during inflammation. It has also been reported that sLRP1 concentrations were higher in severe hypercholesterolemia, and were significantly associated with established pro-atherogenic lipid parameters in two different hypercholesterolemic patients [75]. Moreover, in these individuals, these sLRP1 concentrations were reduced after statin treatment but increased after statin withdrawal. In vitro experiments showed that native LDL, aggregated LDL and VLDL + IDL lipoproteins induced the release of sLRP1 from VSMC cultures, which correlated with an increased sLRP1 in a conditioned medium of coronary atherosclerotic plaque areas extracted from patients [75]. This evidence strongly suggests that sLRP1 is a new potential biomarker for atherosclerosis. 

## 5. Conclusions and Perspectives

**Figure 1:**
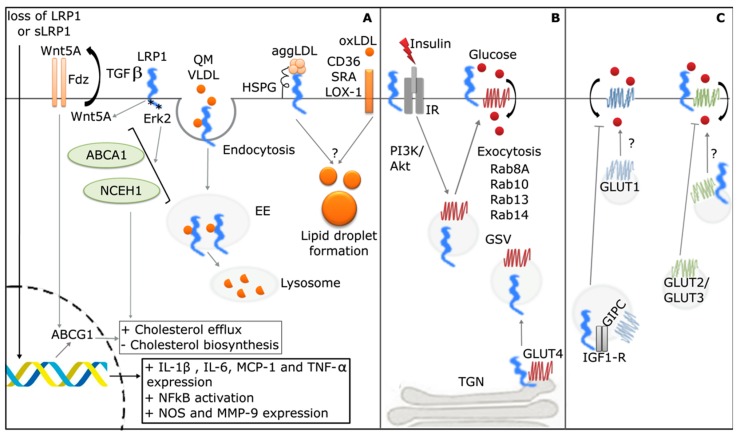
LRP1 role in the lipid metabolisms, glucose homeostasis and inflammation. (**A**) The loss of LRP1 or sLRP1 production can induce pro-inflammatory factors synthesis in macrophages. LRP1 can also regulate the lipid metabolism by endocytosis of cholesterol and triglyceride-rich lipoproteins, such as chylomicrons (QM), very low-density lipoprotein (VLDL) (represented as orange circles) and aggregated low-density lipoprotein (aggLDL) (pink circles) in hepatocytes, vascular smooth muscle cell (VSMCs) and macrophage, and by signaling activation limiting cholesterol intracellular accumulation in fibroblasts; oxidized LDL (oxLDL) is recognized by scavenger receptors (CD36; SRA; and LOX-1) (**B**) LRP1 is essential for the signaling activity of the insulin receptor (IR) in brain and liver, and also in the formation and function of GSVs (GLUT4 storage vesicles) in adipocytes and muscle cells; (**C**) In astrocytes, LRP1 can mediate the GLUT1 trafficking. In neurons and hepatocytes, LRP1 is necessary for GLUT2 and GLUT3 translocation to the plasma membrane. HSPG: Heparan sulfate proteoglycan. SRA: scavenger receptor A. EE: early endosome. NOS: nitric oxide synthase. TGN: *trans*-Golgi network. *: NP-Xy motif. ?: non-characterized mechanism(s).

The role of LRP1 in lipid and glucose metabolism control, as well as in inflammation, is summarized in Figure 1. In Panel A, it can be observed that the function of LRP1 in the regulation of the lipid metabolism may originate from at least two different mechanisms: (i) by signaling activation limiting cholesterol intracellular accumulation, where the cholesterol efflux and inhibition of cholesterol biosynthesis can be regulated by: (a) the extracellular α subunit of LRP1, which mediates TGF-β-induced enhancement of Wnt5a, and (b) the intracellular β subunit of LRP1, through its interaction between the distal NPxY motif and Erk2, thereby up-regulating ABCA1 and NCEH1; and (ii) by endocytosis of apoE-mediated uptake of triglyceride-rich lipoprotein remnants (QM and VLDL), in which cholesterol and triglycerides are accumulated in early endosomes (EE) and then degraded by lysosomes. In contrast, LRP1 is returned to the plasma membrane by recycling endosomes (RE). In addition, LRP1 also binds and internalizes aggLDLs through a mechanism that involves HSPG on the cell surface. By means of LRP1-mediated endocytosis, aggLDLs can be accumulated in lysosomes, with cholesterol esters (CEs) being directly transferred to lipid droplet compartments to form foam cells. However, these processes are still not clearly understood.

In Panel B, it is shown how LRP1 can also control glucose homeostasis, with LRP1 translocation playing an essential role in the signaling activity of insulin receptor (IR) on the plasma membrane of brain and liver. In adipocytes and muscle cells, LRP1 plays a key role in the formation and function of vesicles referred to as GSVs (GLUT4 storage vesicles), from which GLUT4 is transported and fused to the plasma membrane by insulin-induced intracellular signaling. In Panel C, it can be seen that LRP1, together with GIPC (GAIP-interacting protein, C terminus), mediate the molecular interaction between IGF1-R and GLUT1, with GLUT1 being retained inside the cell. However, the absence of this molecular interaction leads to GLUT1 translocation to the plasma membrane by an unknown intracellular pathway. In neurons and hepatocytes, LRP1 is necessary for GLUT2 and GLUT3 translocation to the plasma membrane through a non-characterized mechanism(s). 

Finally, LRP1 is an inflammatory mediator that regulates the proinflammatory factors produced in macrophages. In this way, the LRP1 cell loss sLRP1 production by shedding induces proinflammatory factors (interleukin 1 β (IL-1β), interleukin 6 (IL-6) and tumor necrosis factor-α (TNF-α), as well as monocyte chemoattractant protein 1 (MCP-1)) in macrophages. 

The above findings, taken together, suggest a critical role for LRP1 in MetS, which may be considered as a potential tool for diagnosis of this metabolic disorder associated with the development of atherosclerosis, T2DM, and CVD. The loss of LRP1 in tissues regulating the metabolic control of lipids and glucose may indicate a clinical state of tissue steatosis at locations such as the liver, heart, and vascular wall. In addition, LRP1 suppression in macrophages may provide an early diagnosis of inflammatory processes occurring in atherosclerotic lesions. In addition, for this propose, the protein expression of LRP1 in peripheral blood monocytes by flow cytometry assays might be a useful diagnostic tool for extrapolating the LRP1 level in other tissues [3]. Similarly, this LRP1 monocyte level may be inversely correlated with increased concentrations of plasma sLRP1, which occur in patients with cardiometabolic disease [71]. Another possibility, for LRP1 in MetS could be associated with a potential therapeutic use, by taking into account the development of molecular mediators for inducing LRP1 gene expression, similar to that of the PPARγ analog RGZ [24]. Moreover, certain LRP1 ligands, such as α2-macroglobulin and tissue-type plasminogen activator, can attenuate the gene expression of pro-inflammatory factors in macrophages stimulated with lipopolysaccharide (LPS), whereas other LRP1 ligands, including RAP and lactoferrin, can promote macrophage activation, thus mimicking the effect produced by LRP1 macrophage deletion [20]. Finally, new therapeutic strategies using target peptides with identical residue sequences as ligands recognizing the specific binding domains of LRP1 α-subunit, may be also used in order to suppress and/or control inflammatory processes developed during MetS and atherosclerosis. Related to this, the first artificial LRP1-binding peptide with blood-brain barrier permeability has recently been reported [76], which opens important possibilities of applying advanced therapies for MetS using LRP1 as a molecular and cellular target.

## Abbreviations

ABCA1	ATP binding cassette transporter A1
ApoE	Apolipoprotein E
Akt	Protein kinase B (PKB)
Erk2	Serine/threonine kinase
GLUTs	Glucose transporters
GSVs	GLUT4 storage vesicles
HSPG	Heparan sulfate proteoglycan
IGF1-R	Insulin-like growth factor 1-receptor
IR	Insulin receptor
IL-1β	Interleukin 1 β
IL-6	Interleukin 6
LDL	Low-density lipoprotein
LOX-1	Lectin-type oxidized LDL receptor-1
LRP1	Low-density lipoprotein receptor-related protein-1
MAPK	Mitogen-activated protein kinase
MetS	Metabolic syndrome
MCP-1	Monocyte chemoattractant protein 1
PI3K	Phosphatidylinositol-4,5-bisphosphate 3-kinase
TNF-α	Tumor necrosis factor-α
VLDL	Very low-density lipoprotein
VSMC	Vascular smooth muscle cell

## References

[1] Gonias S.L., Campana W.M. (2014). LDL receptor-related protein-1: A regulator of inflammation in atherosclerosis, cancer, and injury to the nervous system. Am. J. Pathol..

[2] Barcelona P.F., Luna J.D., Chiabrando G.A., Juarez C.P., Bhutto I.A., Baba T., McLeod D.S., Sanchez M.C., Lutty G.A. (2010). Immunohistochemical localization of low density lipoprotein receptor-related protein 1 and α2-Macroglobulin in retinal and choroidal tissue of proliferative retinopathies. Exp. Eye Res..

[3] Ferrer D.G., Jaldin-Fincati J.R., Amigone J.L., Capra R.H., Collino C.J., Albertini R.A., Chiabrando G.A. (2014). Standardized flow cytometry assay for identification of human monocytic heterogeneity and LRP1 expression in monocyte subpopulations: Decreased expression of this receptor in nonclassical monocytes. Cytom. Part A.

[4] Strickland D.K., Gonias S.L., Argraves W.S. (2002). Diverse roles for the LDL receptor family. Trends Endocrinol. Metab..

[5] Boucher P., Herz J. (2011). Signaling through LRP1: Protection from atherosclerosis and beyond. Biochem. Pharmacol..

[6] Gonias S.L., Gaultier A., Jo M. (2011). Regulation of the urokinase receptor (uPAR) by LDL receptor-related protein-1 (LRP1). Curr. Pharm. Des..

[7] Barcelona P.F., Jaldin-Fincati J.R., Sanchez M.C., Chiabrando G.A. (2013). Activated α2-macroglobulin induces Muller glial cell migration by regulating MT1-MMP activity through LRP1. FASEB J..

[8] Rabiej V.K., Pflanzner T., Wagner T., Goetze K., Storck S.E., Eble J.A., Weggen S., Mueller-Klieser W., Pietrzik C.U. (2016). Low density lipoprotein receptor-related protein 1 mediated endocytosis of β1-integrin influences cell adhesion and cell migration. Exp. Cell Res..

[9] Ferrer D.G., Dato V.A., Fincati J.R.J., Lorenc V.E., Sanchez M.C., Chiabrando G.A. (2017). Activated α2-Macroglobulin Induces Mesenchymal Cellular Migration of Raw264.7 Cells Through Low-Density Lipoprotein Receptor-Related Protein 1. J. Cell. Biochem..

[10] Liu C.C., Hu J., Tsai C.W., Yue M., Melrose H.L., Kanekiyo T., Bu G. (2015). Neuronal LRP1 regulates glucose metabolism and insulin signaling in the brain. J. Neurosci..

[11] Actis Dato V., Grosso R.A., Sanchez M.C., Fader C.M., Chiabrando G.A. (2018). Insulin-induced exocytosis regulates the cell surface level of low density lipoprotein-related protein-1 in Muller Glial cells. Biochem. J..

[12] Hernandez-Garzon E., Fernandez A.M., Perez-Alvarez A., Genis L., Bascunana P., Fernandez de la Rosa R., Delgado M., Angel Pozo M., Moreno E., McCormick P.J. (2016). The insulin-like growth factor I receptor regulates glucose transport by astrocytes. Glia.

[13] Jaldin-Fincati J.R., Pavarotti M., Frendo-Cumbo S., Bilan P.J., Klip A. (2017). Update on GLUT4 Vesicle Traffic: A Cornerstone of Insulin Action. Trends Endocrinol. Metab..

[14] Sookoian S., Pirola C.J. (2011). Metabolic syndrome: From the genetics to the pathophysiology. Curr. Hypertens. Rep..

[15] Costales P., Fuentes-Prior P., Castellano J., Revuelta-Lopez E., Corral-Rodriguez M.A., Nasarre L., Badimon L., Llorente-Cortes V. (2015). K Domain CR9 of Low Density Lipoprotein (LDL) Receptor-related Protein 1 (LRP1) Is Critical for Aggregated LDL-induced Foam Cell Formation from Human Vascular Smooth Muscle Cells. J. Biol. Chem..

[16] Ding Y., Xian X., Holland W.L., Tsai S., Herz J. (2016). Low-Density Lipoprotein Receptor-Related Protein-1 Protects Against Hepatic Insulin Resistance and Hepatic Steatosis. EBioMedicine.

[17] Au D.T., Strickland D.K., Muratoglu S.C. (2017). The LDL Receptor-Related Protein 1: At the Crossroads of Lipoprotein Metabolism and Insulin Signaling. J. Diabetes Res..

[18] Bogan J.S. (2012). Regulation of glucose transporter translocation in health and diabetes. Annu. Rev. Biochem..

[19] Schaftenaar F., Frodermann V., Kuiper J., Lutgens E. (2016). Atherosclerosis: The interplay between lipids and immune cells. Curr. Opin. Lipidol..

[20] Mantuano E., Brifault C., Lam M.S., Azmoon P., Gilder A.S., Gonias S.L. (2016). LDL receptor-related protein-1 regulates NFκB and microRNA-155 in macrophages to control the inflammatory response. Proc. Natl. Acad. Sci. USA.

[21] Gauthier A., Vassiliou G., Benoist F., McPherson R. (2003). Adipocyte low density lipoprotein receptor-related protein gene expression and function is regulated by peroxisome proliferator-activated receptor γ. J. Biol. Chem..

[22] Ahmadian M., Suh J.M., Hah N., Liddle C., Atkins A.R., Downes M., Evans R.M. (2013). PPARγ signaling and metabolism: The good, the bad and the future. Nat. Med..

[23] Hiatt W.R., Kaul S., Smith R.J. (2013). The cardiovascular safety of diabetes drugs—Insights from the rosiglitazone experience. N. Engl. J. Med..

[24] Rondon-Ortiz A.N., Lino Cardenas C.L., Martinez-Malaga J., Gonzales-Urday A.L., Gugnani K.S., Bohlke M., Maher T.J., Pino-Figueroa A.J. (2017). High Concentrations of Rosiglitazone Reduce mRNA and Protein Levels of LRP1 in HepG2 Cells. Front. Pharmacol..

[25] Vucinic N., Stokic E., Djan I., Obreht D., Velickovic N., Stankov K., Djan M. (2017). The LRP1 Gene Polymorphism is associated with Increased Risk of Metabolic Syndrome Prevalence in the Serbian Population. Balkan J. Med. Genet..

[26] Delgado-Lista J., Perez-Martinez P., Solivera J., Garcia-Rios A., Perez-Caballero A.I., Lovegrove J.A., Drevon C.A., Defoort C., Blaak E.E., Dembinska-Kieć A. (2014). Top single nucleotide polymorphisms affecting carbohydrate metabolism in metabolic syndrome: From the LIPGENE study. J. Clin. Endocrinol. Metab..

[27] Mahley R.W., Huang Y. (2007). Atherogenic remnant lipoproteins: Role for proteoglycans in trapping, transferring, and internalizing. J. Clin. Investig..

[28] May P., Bock H.H., Herz J. (2003). Integration of endocytosis and signal transduction by lipoprotein receptors. Sci. Signal..

[29] Rensen P.C., Jong M.C., van Vark L.C., van der Boom H., Hendriks W.L., van Berkel T.J., Biessen E.A., Havekes L.M. (2000). Apolipoprotein E is resistant to intracellular degradation in vitro and in vivo. Evidence for retroendocytosis. J. Biol. Chem..

[30] Laatsch A., Panteli M., Sornsakrin M., Hoffzimmer B., Grewal T., Heeren J. (2012). Low density lipoprotein receptor-related protein 1 dependent endosomal trapping and recycling of apolipoprotein E. PLoS ONE.

[31] Lillis A.P., Mikhailenko I., Strickland D.K. (2005). Beyond endocytosis: LRP function in cell migration, proliferation and vascular permeability. J. Thromb. Haemost..

[32] Heeren J., Beisiegel U., Grewal T. (2006). Apolipoprotein E recycling: Implications for dyslipidemia and atherosclerosis. Arterioscler. Thromb. Vasc. Biol..

[33] Kowal R.C., Herz J., Goldstein J.L., Esser V., Brown M.S. (1989). Low density lipoprotein receptor-related protein mediates uptake of cholesteryl esters derived from apoprotein E-enriched lipoproteins. Proc. Natl. Acad. Sci. USA.

[34] Beisiegel U., Weber W., Ihrke G., Herz J., Stanley K.K. (1989). The LDL-receptor-related protein, LRP, is an apolipoprotein E-binding protein. Nature.

[35] Beisiegel U., Weber W., Bengtsson-Olivecrona G. (1991). Lipoprotein lipase enhances the binding of chylomicrons to low density lipoprotein receptor-related protein. Proc. Natl. Acad. Sci. USA.

[36] Heeren J., Niemeier A., Merkel M., Beisiegel U. (2002). Endothelial-derived lipoprotein lipase is bound to postprandial triglyceride-rich lipoproteins and mediates their hepatic clearance in vivo. J. Mol. Med..

[37] Laatsch A., Merkel M., Talmud P.J., Grewal T., Beisiegel U., Heeren J. (2009). Insulin stimulates hepatic low density lipoprotein receptor-related protein 1 (LRP1) to increase postprandial lipoprotein clearance. Atherosclerosis.

[38] Zheng X.Y., Yu B.L., Xie Y.F., Zhao S.P., Wu C.L. (2017). Apolipoprotein A5 regulates intracellular triglyceride metabolism in adipocytes. Mol. Med. Rep..

[39] Lillis A.P., Muratoglu S.C., Au D.T., Migliorini M., Lee M.J., Fried S.K., Mikhailenko I., Strickland D.K. (2015). LDL Receptor-Related Protein-1 (LRP1) Regulates Cholesterol Accumulation in Macrophages. PLoS ONE.

[40] Xian X., Ding Y., Dieckmann M., Zhou L., Plattner F., Liu M., Parks J.S., Hammer R.E., Boucher P., Tsai S. (2017). LRP1 integrates murine macrophage cholesterol homeostasis and inflammatory responses in atherosclerosis. eLife.

[41] Fulop T., Khalil A., Larbi A. (2012). The role of elastin peptides in modulating the immune response in aging and age-related diseases. Pathol. Biol..

[42] Samouillan V., Dandurand J., Nasarre L., Badimon L., Lacabanne C., Llorente-Cortes V. (2012). Lipid loading of human vascular smooth muscle cells induces changes in tropoelastin protein levels and physical structure. Biophys. J..

[43] Samouillan V., Revuelta-Lopez E., Dandurand J., Nasarre L., Badimon L., Lacabanne C., Llorente-Cortés V. (2014). Cardiomyocyte intracellular cholesteryl ester accumulation promotes tropoelastin physical alteration and degradation: Role of LRP1 and cathepsin S. Int. J. Biochem. Cell Biol..

[44] El Asmar Z., Terrand J., Jenty M., Host L., Mlih M., Zerr A., Justiniano H., Matz R.L., Boudier C., Scholler E. (2016). Convergent Signaling Pathways Controlled by LRP1 (Receptor-related Protein 1) Cytoplasmic and Extracellular Domains Limit Cellular Cholesterol Accumulation. J. Biol. Chem..

[45] Alique M., Luna C., Carracedo J., Ramirez R. (2015). LDL biochemical modifications: A link between atherosclerosis and aging. Food Nutr. Res..

[46] Yang T.C., Chang P.Y., Kuo T.L., Lu S.C. (2017). Electronegative L5-LDL induces the production of G-CSF and GM-CSF in human macrophages through LOX-1 involving NF-κB and ERK2 activation. Atherosclerosis.

[47] Yang T.C., Chang P.Y., Lu S.C. (2017). L5-LDL from ST-elevation myocardial infarction patients induces IL-1β production via LOX-1 and NLRP3 inflammasome activation in macrophages. Am. J. Physiol. Heart Circ. Physiol..

[48] Saneipour M., Ghatreh-Samani K., Heydarian E., Farrokhi E., Abdian N. (2015). Adiponectin inhibits oxidized low density lipoprotein-induced increase in matrix metalloproteinase 9 expression in vascular smooth muscle cells. ARYA Atheroscler..

[49] Lin J., Zhou S., Zhao T., Ju T., Zhang L. (2016). TRPM7 channel regulates ox-LDL-induced proliferation and migration of vascular smooth muscle cells via MEK-ERK pathways. FEBS Lett..

[50] Boren J., Williams K.J. (2016). The central role of arterial retention of cholesterol-rich apolipoprotein-B-containing lipoproteins in the pathogenesis of atherosclerosis: A triumph of simplicity. Curr. Opin. Lipidol..

[51] Ke L.Y., Chan H.C., Chan H.C., Kalu F.C.U., Lee H.C., Lin I.L., Jhuo S.J., Lai W.T., Tsao C.R., Sawamura T. (2017). Electronegative Low-Density Lipoprotein L5 Induces Adipose Tissue Inflammation Associated With Metabolic Syndrome. J. Clin. Endocrinol. Metab..

[52] Dai Y., Su W., Ding Z., Wang X., Mercanti F., Chen M., Raina S., Mehta J.L. (2013). Regulation of MSR-1 and CD36 in macrophages by LOX-1 mediated through PPAR-γ. Biochem. Biophys. Res. Commun..

[53] Ganesan R., Henkels K.M., Wrenshall L.E., Kanaho Y., Paolo G.D., Frohman M.A., Gomez-Cambronero J. (2018). Oxidized LDL phagocytosis during foam cell formation in atherosclerotic plaques relies on a PLD2-CD36 functional interdependence. J. Leukoc. Biol..

[54] Llorente-Cortes V., Otero-Vinas M., Badimon L. (2002). Differential role of heparan sulfate proteoglycans on aggregated LDL uptake in human vascular smooth muscle cells and mouse embryonic fibroblasts. Arterioscler. Thromb. Vasc. Biol..

[55] Dissmore T., Seye C.I., Medeiros D.M., Weisman G.A., Bradford B., Mamedova L. (2016). The P2Y2 receptor mediates uptake of matrix-retained and aggregated low density lipoprotein in primary vascular smooth muscle cells. Atherosclerosis.

[56] Mueckler M., Thorens B. (2013). The SLC2 (GLUT) family of membrane transporters. Mol. Aspects Med..

[57] Huang G., Buckler-Pena D., Nauta T., Singh M., Asmar A., Shi J., Kim J.Y., Kandror K.V. (2013). Insulin responsiveness of glucose transporter 4 in 3T3-L1 cells depends on the presence of sortilin. Mol. Biol. Cell.

[58] Thorens B. (2015). GLUT2, glucose sensing and glucose homeostasis. Diabetologia.

[59] Jedrychowski M.P., Gartner C.A., Gygi S.P., Zhou L., Herz J., Kandror K.V., Pilch P.F. (2010). Proteomic analysis of GLUT4 storage vesicles reveals LRP1 to be an important vesicle component and target of insulin signaling. J. Biol. Chem..

[60] Pan X., Zaarur N., Singh M., Morin P., Kandror K.V. (2017). Sortilin and retromer mediate retrograde transport of Glut4 in 3T3-L1 adipocytes. Mol. Biol. Cell.

[61] Muratoglu S.C., Belgrave S., Lillis A.P., Migliorini M., Robinson S., Smith E., Zhang L., Strickland D.K. (2011). Macrophage LRP1 suppresses neo-intima formation during vascular remodeling by modulating the TGF-β signaling pathway. PLoS ONE.

[62] Baitsch D., Bock H.H., Engel T., Telgmann R., Muller-Tidow C., Varga G., Bot M., Herz J., Robenek H., von Eckardstein A. (2011). Apolipoprotein E induces antiinflammatory phenotype in macrophages. Arterioscler. Thromb. Vasc. Biol..

[63] Overton C.D., Yancey P.G., Major A.S., Linton M.F., Fazio S. (2007). Deletion of macrophage LDL receptor-related protein increases atherogenesis in the mouse. Circ. Res..

[64] Zhu L., Giunzioni I., Tavori H., Covarrubias R., Ding L., Zhang Y., Ormseth M., Major A.S., Stafford J.M., Linton M.F. (2016). Loss of Macrophage Low-Density Lipoprotein Receptor-Related Protein 1 Confers Resistance to the Antiatherogenic Effects of Tumor Necrosis Factor-α Inhibition. Arterioscler. Thromb. Vasc. Biol..

[65] Omar A., Chatterjee T.K., Tang Y., Hui D.Y., Weintraub N.L. (2014). Proinflammatory phenotype of perivascular adipocytes. Arterioscler. Thromb. Vasc. Biol..

[66] Brown N.K., Zhou Z., Zhang J., Zeng R., Wu J., Eitzman D.T., Chen Y.E., Chang L. (2014). Perivascular adipose tissue in vascular function and disease: A review of current research and animal models. Arterioscler. Thromb. Vasc. Biol..

[67] Konaniah E.S., Kuhel D.G., Basford J.E., Weintraub N.L., Hui D.Y. (2017). Deficiency of LRP1 in Mature Adipocytes Promotes Diet-Induced Inflammation and Atherosclerosis-Brief Report. Arterioscler. Thromb. Vasc. Biol..

[68] Yancey P.G., Blakemore J., Ding L., Fan D., Overton C.D., Zhang Y., Linton M.F., Fazio S. (2010). Macrophage LRP-1 controls plaque cellularity by regulating efferocytosis and Akt activation. Arterioscler. Thromb. Vasc. Biol..

[69] Subramanian M., Hayes C.D., Thome J.J., Thorp E., Matsushima G.K., Herz J., Farber D.L., Liu K., Lakshmana M., Tabas I. (2014). An AXL/LRP-1/RANBP9 complex mediates DC efferocytosis and antigen cross-presentation in vivo. J. Clin. Investig..

[70] Mueller P.A., Zhu L., Tavori H., Huynh K., Giunzioni I., Stafford J.M., Linton M.F., Fazio S. (2018). Deletion of Macrophage Low-Density Lipoprotein Receptor-Related Protein 1 (LRP1) Accelerates Atherosclerosis Regression and Increases CCR7 Expression in Plaque Macrophages. Circulation.

[71] Gonzalo-Calvo D., Colom C., Vilades D., Rivas-Urbina A., Moustafa A.H., Perez-Cuellar M., Sánchez-Quesada J.L., Pérez A., LLorente-Cortes V. (2018). Soluble LRP1 is an independent biomarker of epicardial fat volume in patients with type 1 diabetes mellitus. Sci. Rep..

[72] De Gonzalo-Calvo D., Vilades D., Nasarre L., Carreras F., Leta R., Garcia-Moll X., Llorente-Cortes V. (2016). Circulating levels of soluble low-density lipoprotein receptor-related protein 1 (sLRP1) as novel biomarker of epicardial adipose tissue. Int. J. Cardiol..

[73] Von Arnim C.A., Kinoshita A., Peltan I.D., Tangredi M.M., Herl L., Lee B.M., Spoelgen R., Hshieh T.T., Ranganathan S., Battey F.D. (2005). The low density lipoprotein receptor-related protein (LRP) is a novel β-secretase (BACE1) substrate. J. Biol. Chem..

[74] Gorovoy M., Gaultier A., Campana W.M., Firestein G.S., Gonias S.L. (2010). Inflammatory mediators promote production of shed LRP1/CD91, which regulates cell signaling and cytokine expression by macrophages. J. Leukoc. Biol..

[75] De Gonzalo-Calvo D., Cenarro A., Martinez-Bujidos M., Badimon L., Bayes-Genis A., Ordonez-Llanos J., Civeira F., Llorente-Cortés V. (2015). Circulating soluble low-density lipoprotein receptor-related protein 1 (sLRP1) concentration is associated with hypercholesterolemia: A new potential biomarker for atherosclerosis. Int. J. Cardiol..

[76] Sakamoto K., Shinohara T., Adachi Y., Asami T., Ohtaki T. (2017). A novel LRP1-binding peptide L57 that crosses the blood brain barrier. Biochem. Biophys. Rep..

